# Optimizing expression of the pregnancy malaria vaccine candidate, VAR2CSA in *Pichia pastoris*

**DOI:** 10.1186/1475-2875-8-143

**Published:** 2009-06-29

**Authors:** Marion Avril, Marianne J Hathaway, Megan M Cartwright, Severin O Gose, David L Narum, Joseph D Smith

**Affiliations:** 1 Seattle Biomedical Research Institute, 307 Westlake Ave N, Suite 500, Seattle Washington, 98109-5219, USA; 2 Malaria Vaccine Development Branch, National Institute of Allergy and Infectious Diseases, National Institutes of Health, Twinbrook I, 5640 Fishers Lane, Rockville, Maryland 20852, USA

## Abstract

**Background:**

VAR2CSA is the main candidate for a vaccine against pregnancy-associated malaria, but vaccine development is complicated by the large size and complex disulfide bonding pattern of the protein. Recent X-ray crystallographic information suggests that domain boundaries of VAR2CSA Duffy binding-like (DBL) domains may be larger than previously predicted and include two additional cysteine residues. This study investigated whether longer constructs would improve VAR2CSA recombinant protein secretion from *Pichia pastoris *and if domain boundaries were applicable across different VAR2CSA alleles.

**Methods:**

VAR2CSA sequences were bioinformatically analysed to identify the predicted C11 and C12 cysteine residues at the C-termini of DBL domains and revised N- and C-termimal domain boundaries were predicted in VAR2CSA. Multiple construct boundaries were systematically evaluated for protein secretion in *P. pastoris *and secreted proteins were tested as immunogens.

**Results:**

From a total of 42 different VAR2CSA constructs, 15 proteins (36%) were secreted. Longer construct boundaries, including the predicted C11 and C12 cysteine residues, generally improved expression of poorly or non-secreted domains and permitted expression of all six VAR2CSA DBL domains. However, protein secretion was still highly empiric and affected by subtle differences in domain boundaries and allelic variation between VAR2CSA sequences. Eleven of the secreted proteins were used to immunize rabbits. Antibodies reacted with CSA-binding infected erythrocytes, indicating that *P. pastoris *recombinant proteins possessed native protein epitopes.

**Conclusion:**

These findings strengthen emerging data for a revision of DBL domain boundaries in *var*-encoded proteins and may facilitate pregnancy malaria vaccine development.

## Background

Pregnancy-associated malaria (PAM) is an important cause of maternal anaemia, stillbirth, and delivery of low birth weight children in malaria endemic regions [1]. PAM is characterized by the selective accumulation of *Plasmodium falciparum*-infected erythrocytes (IEs) in the placental microvasculature mediated by chondroitin sulphate A (CSA) [2]. VAR2CSA is an unusually conserved member of the *P. falciparum *erythrocyte membrane protein 1 (PfEMP1) family, which is transcriptionally upregulated in CSA-binding and placental isolates and binds CSA [3-10]. Antibodies to VAR2CSA are developed in a gender specific manner [11,12] and correlate with protection from PAM disease [7] making it the most promising vaccine candidate against placenta malaria, but vaccine development is complicated by protein size (~350 kDa) and polymorphism [13].

VAR2CSA contains six different Duffy-binding-like (DBL) domains and additional interdomain regions. DBL are adhesion modules found in both parasite ligands used for erythrocyte invasion and PfEMP1 proteins utilized by infected erythrocytes to sequester from blood circulation and avoid spleen-dependent killing mechanisms [14,15]. The DBL structure has been solved from three different proteins, the erythrocyte invasion ligands, *Plasmodium knowlesi *α and EBA-175, as well as the VAR2CSA DBL3 domain [5,8,16,17]. DBL domains have highly similar protein folds, despite limited sequence similarity, and are characterized by conserved disulfide bonds. Although significant progress has been made in heterologous production of DBL recombinant proteins [18-24], proteins containing multiple disulfide bonds are generally considered much more challenging to produce than cytoplasmic proteins posing challenges to pregnancy malaria vaccine development. Furthermore, understanding of optimal domain boundaries for VAR2CSA immunogens remains incomplete.

Because of its large size it has not been technically possible to express the complete VAR2CSA extracellular region, and instead vaccine development has initially focused on expressing the individual DBL domains. There are numerous standardized systems for protein expression which offer a variety of benefits and disadvantages, such as *Escherichia coli*, baculovirus infected insect cells, and the methyltrophic yeast *Pichia pastoris*. While general protein expression is often faster in *E. coli *than *P. pastoris*, bacteria lack the machinery to produce properly folded proteins where the tertiary structure is highly dependent on disulfide bonds. *E. coli *production of disulfide-rich proteins frequently require multiple post-production steps, which must be empirically determined and lower effective protein yield [25]. The major advantage of *P. pastoris *or baculovirus infected insect cells over *E. coli *is that the yeast or insect cell secretory systems provide the necessary redox environment and co-factors to enhance the correct folding, solubility, and disulfide bonds present in many vaccine candidates. All three expression systems are being investigated for VAR2CSA vaccine development [18,19,21,26], but only a subset of the DBL domains have been analysed in *E. coli *and *P. pastoris *because of difficulty in producing some recombinant proteins.

Recently, the crystal structure has been solved for the VAR2CSA DBL3 domain [5,8]. This structure indicates that PfEMP1-type DBL domains may be longer than previously appreciated and contain three additional cysteine residues, one at the N-terminus and two at the C-terminus, which are involved in disulfide bonds. As the initial and final two cysteine residues were missing in previous recombinant proteins produced in *P. pastoris *[18] and *E. coli *[21], it was possible that domain truncation may affect protein conformation, stability, surface antigenicity, and/or the ability to secrete the protein from *P. pastoris*. In this study, the new structural insights into DBL disulfide structure [5,8,16,17] were used to predict disulfide bonds in VAR2CSA leading to revised DBL domain boundaries. Based on the new domain boundaries, multiple VAR2CSA constructs were evaluated in *P. pastoris *to investigate the role of additional cysteine residues in recombinant protein secretion and to determine whether construct boundaries that worked well with one VAR2CSA sequence were applicable across different VAR2CSA alleles.

## Methods

### Design of DBL synthetic genes

Synthetic genes were constructed by (Genscript Corporation, Piscatway, NJ, USA) and codon optimized for *P. pastoris *expression. All N-glycosylation sites were removed by converting asparagine to glutamine and DNA sequence carrying more than five adenine nucleotides in a row was mutated to avoid any premature termination without changing coding features.

### Recombinant protein production in *P. pastoris*

VAR2CSA constructs with a His_6_-tag on the C-terminus were amplified from synthetic genes and cloned into the pPIC9K vector adjacent to an N-terminal α-factor secretion signal (Invitrogen, USA). Constructs were digested with *SacI *and electroporated into *P. pastoris *strain, GS115. The transformation resulted in DNA inserted at the AOX1 locus generating a His+ Mut+ phenotype. To screen for protein production, 10–20 yeast clones per construct were grown overnight in 5ml YPD (1% yeast extract, 2% tryptone, 20% glucose) at 30°C and then 0.2 ml volume of cells was transferred to 5 ml buffered complex medium (BM) (1% yeast extract, 2% peptone, 1% yeast nitrogen base, 1 M potassium phosphate buffer pH 6.0) plus 2% glycerol (BMG) and grown for an additional 24 h at 30°C with shaking at 250 rpm. For protein induction, cultures were shifted to BM plus 0.5% methanol (BMM) and grown for two to five days at 20°C with shaking at 250 rpm. *Pichia pastoris *recombinant proteins were analysed in 4–20% SDS-PAGE gels under reduced or non-reduced conditions (Invitrogen, USA). Gels were stained with Gel Code Blue Reagent or transferred to a nitrocellulose membrane and detected by Western Blot using anti-His tag antibodies (Invitrogen, USA). For scaled-up production, positive secreting *P. pastoris *clones were grown in a 2 L shaker flask using the same methodology as above but with 0.9 L YPD, 0.3 L BMG and 0.3 L BMM respectively and a methanol concentration of 0.5%, 1% or 3%. HIS-tagged recombinant proteins were harvested 48 h or 120 h post-induction using nickel resin or cobalt-nitrilotriacetic acid-agarose (Sigma, USA) on an Econo-Pac chromatography column (Biorad, USA). Proteins were eluted in 200 mM imidazole following manufacturer's instructions and fractions containing the protein were detected by Western Blot, pooled together and dialyzed via buffer exchange with 1× PBS. Protein concentrations were determined by Bradford Assay (Biorad, 500-0205, USA). The identity of each recombinant protein was confirmed by mass spectrometry analyses. Briefly, the protein sample was digested with trypsin, and the peptides were analysed by nano-LC-MS/MS using Sequest algorithm. Purified proteins were stored at -80°C in 1× PBS prior to immunization.

### Immunization and serological analysis of VAR2CSA recombinant proteins

Immunizations were performed at R&R Rabbitry (Washington, USA) according to animal immunization guidelines. In brief, rabbits received 25–50 μg recombinant protein in complete Freund's adjuvant for first immunization and were boosted four times with the same amount of recombinant protein in incomplete Freund's adjuvant. Preimmune and immune rabbit sera were analysed by flow cytometry on the homologous CSA-binding infected erythrocytes or as a negative control sera were screened on a non-CSA binding parasite line called A4ultra, which expresses a different PfEMP1 variant from VAR2CSA. The A4ultra parasite line expresses a PfEMP1 protein called A4var. Expression of the A4var PfEMP1 variant was maintained in A4ultra-infected erythrocytes by periodic selection with mAb BC6 that is specific to the A4var PfEMP1 protein [27]. The various CSA-binding lines were maintained by CSA-selection *in vitro*. Prior to flow cytometry, *var2csa *transcription was validated by qRT-PCR using universal *var2csa *primers and compared to a housekeeping control gene, as described [18]. For flow cytometry, mature-stage IEs were washed in PBS and resuspended in a 0.1 ml volume of PBS-1% bovine serum albumin containing a 1/25 dilution of rabbit sera preadsorbed on uninfected erythrocytes. Bound antibodies were detected with Alexa Fluor 488-conjugated goat anti-rabbit IgG. Infected erythrocytes were detected using ethidium bromide. Samples were analysed on an LSRII (BD, USA).

## Results and discussion

### Reevaluation of domain boundaries in VAR2CSA

**Figure 1:**
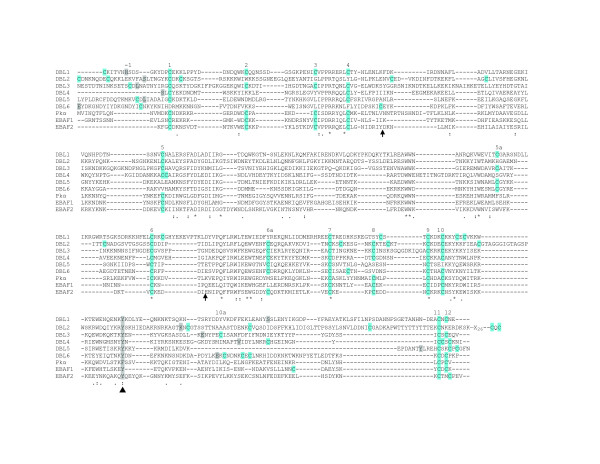
**DBL sequence alignment**. The six DBL domains in VAR2CSA are compared to erythrocyte binding proteins. Cysteine residues are highlighted in blue shading and numbered -1 to 12 based on conservation between erythrocyte binding proteins. The end of the sequence homology between PfEMP1 and erythrocyte binding-type DBL domains is indicated with an arrowhead and grey shading of the final F/Y residue. The S1, S2 and S3 subdomains boundaries are indicated by arrows. The start and end residues in the original VAR2CSA DBL constructs are indicated by grey shading. There is a 26 amino acid spacer before the CXC motif in the DBL2 domain. Accession numbers are IT4-VAR2CSA (AAQ73926), EBA-175 (MAL7P1.176), and *P. knowlesi *α (M90466).

**Figure 2:**
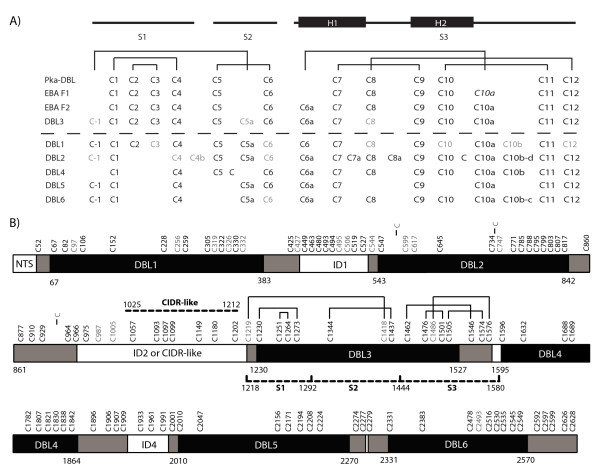
**Reevaluation of VAR2CSA domain boundaries**. A) The disulfide bond structure in four solved DBL domains [5,8,16,17] is shown above the dashed line. An unpaired C10a cysteine residue in EBA175 F1 is italicized. Below the dashed line is the predicted disulfide bonds in VAR2CSA domains. Black cysteines are invariant, grey cysteines were present in more than 50% of sequences from a comparison of 19 *var2csa *sequences [13]. The location of S1, S2, and S3 subdomains, including anti-parallel α-helical elements in the S3 subdomain are shown. B) VAR2CSA protein schematic showing DBL domains and interdomain (ID) regions. The original DBL domain boundaries are shown in black and numbered according to IT4-VAR2CSA (AAQ73926). Revised domain boundaries including predicted C11 and C12 cysteines are shown in grey. The DBL6 domain has two CX_1–2_C motifs, either of which may mark the domain end. Cysteine residues are numbered from the protein start and colored black or grey as described above. The three unnumbered cysteine residues are not present in the IT4 VAR2CSA sequence, but were present in more than 50% of VAR2CSA sequences. The location of disulfide bonds in the solved DBL3 domain [5,8] and the predicted CIDR-like domain [30,31] are indicated.

Based on initial sequence criteria [28,29], DBL domains in PfEMP1 proteins were predicted to begin at a cysteine residue (C1) and end shortly after the tenth cysteine residue (C10) at a (W/Y)X_6_(Y/F) motif (Figure 1). However, these domain boundaries may need to be revised because DBL domains in erythrocyte invasion ligands have two or three additional cysteine residues after C10, and these were found to make conserved disulfide bounds with internal cysteine residues when the DBL structures were solved from the EBA175 F1 and F2 DBL domains and from the *P. knowlesi *α protein (Figure 2) [16,17]. Furthermore, the CIDR1 structure has been solved from one PfEMP1 protein and this led to the prediction that C11 and C12 residues are present in PfEMP1 DBL1 domains, but may have been missed due to lack of sequence homology in the region between C10–C12 (Figure 1) [30]. This prediction has been confirmed for the VAR2CSA DBL3 domain [5,8] demonstrating that the C-terminal disulfide bonding pattern has been conserved in diverse DBL domains (Figure 2). In addition, the VAR2CSA DBL3 domain had an additional disulfide bond at the N-terminus, which was not present in erythrocyte invasion ligands, between a cysteine residue at the minus one position C(-1) and an additional cysteine residue between C5 and C6, referred to as C5a (Figure 1). Therefore, it may be necessary to extend the N- and C-terminal boundaries in VAR2CSA DBL domains.

To predict the disulfide bonding pattern of the VAR2CSA DBL domains, sequence alignments of the six VAR2CSA DBL domains were compared to the three solved DBL domains from erythrocyte invasion ligands (Figure 1). DBL domains from erythrocyte invasion ligands have twelve to fourteen conserved cysteine residues (Figure 2). Sequence analysis indicates that many of these same cysteine residues are present in VAR2CSA DBL domains (Figure 1), but that each VAR2CSA domain will have slightly distinct disulfide bonding pattern because of gain or loss of cysteine residues. For instance, the C5a residue has the motif WW X_7 _W X_4 _C. This motif is present in four of the six VAR2CSA DBL domains, plus the DBL2 domain had a cysteine residue that came 13 amino acids after the tryptophan residue (Figure 1). This suggests that the VAR2CSA DBL1, DBL5, DBL6, and possibly DBL2 domains may make a C(-1) to C5a disulfide bond, similar to VAR2CSA DBL3 (Figure 2) and that the N-terminal boundary of these domains may need to be extended upstream by at least one cysteine residue to account for this disulfide bond (grey bars in Figure 2). Consistent with this idea, the DBL4 domain does not contain a C5a residue (Figure 1) and the region upstream of DBL4 does not contain a free cysteine residue for disulfide bonding (Figure 2). Based on the IT4 VAR2CSA sequence (accession number AAQ73926) and counting amino acids from the beginning of the protein, this would imply that DBL1 begins at residue C52, DBL2 at C527 or C544, DBL5 at C2001, and DBL6 at C2279 (Figure 2). All of these cysteine residues were 100% conserved in a global-wide comparison of VAR2CSA sequences [13], except for C544, which is why the 100% conserved C527 residue is an alternate potential beginning of the DBL2 domain. Of interest, the C(-1) and C5a residues in DBL3 are not 100% conserved in all VAR2CSA sequences although this disulfide bond was present in the IT4 VAR2CSA DBL3 crystal structure [5]. This may mean that C5a is sometimes a free cysteine since the next upstream cysteine residue (C1202) is predicted to participate in a CIDR-like fold [30,31]. As most PfEMP1-type DBL domains have a WW X_7 _W X_4 _C [28] this may imply that the C(-1) to C5a disulfide bond is under selection in PfEMP1 proteins and that N-terminal domain boundaries will need to be shifted upstream by one cysteine residue.

At the C-terminus, the final two cysteine residues in DBL domains generally have the motif CX_1–2_C [5,8,16,17]. However, in the four solved DBL structures the C11 and C12 cysteines are located between 43–55 amino acids downstream of the previous DBL domain boundary predicted by sequence homology in PfEMP1 proteins (Figure 1), and have been predicted to be up to 80–100 residues downstream for the PfEMP1 DBL1 domain [30]. Inspection of a VAR2CSA sequence alignment indicates that five of the six VAR2CSA DBL domains have a CX_1–2_C motif located between 28–80 amino acids downstream of the previous domain boundaries and DBL2 domain had one 124 residues away (Figure 1, grey extensions in Figure 2). In addition, the DBL6 domain had two potential CX_1–2_C motifs located near each other (Figure 2). All of the CX_1–2_C motifs were 100% conserved or missing from at most one parasite isolate in a global sampling of VAR2CSA sequences [13] suggesting they may be under structural or functional selection. While DBL2 is potentially longer than other VAR2CSA domains, the extended DBL2 boundary is supported by homology modeling predictions that the ID2 region contains a CIDR-like fold between residues 1025 to 1212 [30,31]. Thus, the first part of ID2 may fold as part of DBL2 and second part assume a CIDR-like fold. Based on amino acid numbering in the IT4 VAR2CSA sequence this implies the DBL1 ends after C427, DBL2 after C966, DBL3 after C1576, DBL4 after C1909, DBL5 after C2277, and DBL6 after C2599 or C2628, although the second CX_1–2_C motif is more likely.

Of the C-terminus predictions, the most difficult domains to unambiguously assign C11 and C12 cysteine residues were the DBL1, DBL2, and DBL4 because these were all followed by larger interdomain regions with multiple cysteine residues (Figure 2). However, the fact that the C11 and C12 disulfide bonding partner are conserved in every domain except DBL5 also supports extending domain boundaries. If C8–C12 and C10–C11 disulfide bonds were no longer present, then it might have been expected that "pairwise loss" may have occurred, as happened to the C2–C3 bond in DBL2, DBL4, DBL5 and DBL6 (Figure 2). Furthermore, at least five of the VAR2CSA domains appear to have a C6a residue that may make a C6a–C10a disulfide bond similar to VAR2CSA-DBL3 and EBAF2 (Figure 2) [8,17]. The remaining domain, DBL1, has a cysteine residue between C5 and C6, but it is not in the typical C6a position (Figure 1), so it is more difficult to predict a disulfide bond interaction. Taken together, the conservation of CX_1–2_C motifs and retention of C6a, C8, and C10 cysteines residues support that VAR2CSA DBL domains have the classic subdomain 3 disulfide bonding pattern (Figure 2). Although only two disulfide bonds (C1–C4 and C7–C9) are predicted to be present in all of the VAR2CSA domains (Figure 2), this analysis suggests that the N-terminal and C-terminal boundaries of the DBL domains need to be extended in VAR2CSA. After making these adjustments, there are still a few remaining cysteine residues in the ID1 and ID4 regions (Figure 2). It remains to be determined whether these regions fold independently or as part of adjacent DBL domains.

### Evaluation of longer VAR2CSA construct boundaries

**Figure 3:**
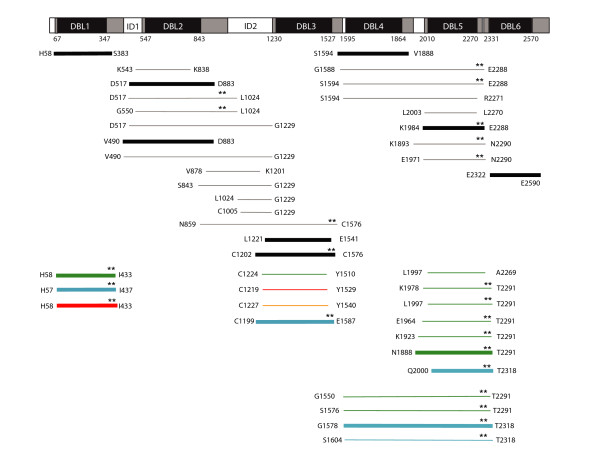
**VAR2CSA construct boundaries**. VAR2CSA protein schematic showing original domain boundaries in black and revised domain boundaries in grey. The start and stop positions for each construct are shown; IT4-*var2csa *allele in black, 3D7-*var2csa *in green, 7G8-*var2csa *in blue, Dd2-*var2csa *in red and Pc49-*var2csa *in orange. Thick bars indicates the recombinant protein was secreted by *P. pastoris*, thin bars means it was not. The presence of predicted C11 and C12 cysteine residues at the C-terminus of constructs is indicated by two asterisks.

**Figure 4:**
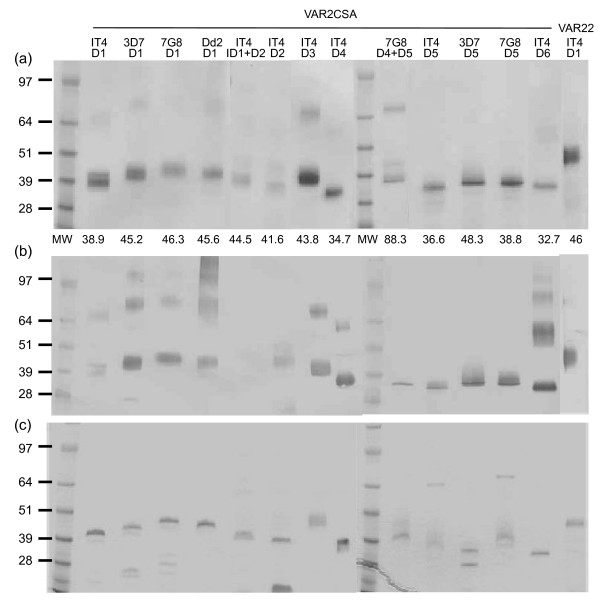
**VAR2CSA recombinant proteins secreted by *P. pastoris***. 1 μg of purified *P. pastoris *recombinant proteins were analysed in 4–20% SDS-PAGE gels under non-reduced (A, B) or reduced (C) conditions. Proteins were stained with Gel Code Blue Reagent in (A) and (C) or were detected by Western blot with an anti-His Tag antibody (B).

Previously [18], four of the six VAR2CSA DBL domains have been secreted from *P. pastoris*, but these constructs generally began ~1–9 amino acids upstream of C1 and ended ~15–35 residues after the (W/Y)X_6_(Y/F) motif (Figure 1), and therefore would be truncated compared to the new domain boundaries. The inability to secrete some VAR2CSA domains in *P. pastoris *may be related to protein mis-folding and ER quality control mechanisms since truncated domains would possess free thiols that do not have their normal bridging partner. To investigate whether longer construct boundaries would improve recombinant protein expression, new VAR2CSA constructs were investigated starting with the DBL2, DBL3, and DBL5 domains that were poorly or not secreted from *P. pastoris *[18]. Initially, DBL3 was chosen because the structure was solved and disulfide bonding pattern confirmed [5,8]. In the original DBL3 construct L1221-E1541, the final three cysteine residues (C10a, C11, and C12) were missing (Figures 2 and 3). This protein was poorly secreted, but expression could be improved by co-transforming with a plasmid overexpressing the protein disulfide isomerase and biofermentation [18]. The same truncated domain boundaries also did not work for DBL3 domains from three other VAR2CSA alleles (Figure 3). However, IT4-DBL3 protein secretion was improved by including the complete S3 subdomain owHo(C1202–C1576) and a full-length 7G8-DBL3 recombinant protein (C1199-E1587) was also highly expressed (Table 1). Under non-reducing conditions, the majority of DBL3 recombinant protein appeared to run as a monomer, although smaller amounts of higher, dimeric-sized protein forms were visible by Western (Figure 4). Therefore, full-length DBL3 constructs were better secreted than truncated domains, and the equivalent construct boundaries worked for both IT4 and 7G8.

**Table 1:** Expression of PfEMP1 domains in *P. pastoris*

Gene	Domain	Parasite strain	construct	MW^1^	Protein Secreted	IE surface reactivity^2^
var2CSA	DBL1	IT/FCR3 ^3^	H58 – S383	38.9	++	yes
var2CSA	DBL1	3D7	H58 – I433	45.2	++	yes
var2CSA	DBL1	7G8	H57-I437	46.3	++	yes
var2CSA	DBL1	Dd2	H57-I433	45.6	++	yes
var2CSA	ID1-DBL2	IT/FCR3	V490 – D883	44.5	+	nt
var2CSA	DBL2	IT/FCR3 ^3^	K543 – K838	36.1	-	nt
var2CSA	DBL2	IT/FCR3	D517 – D883	41.6	+	nt
var2CSA	DBL2	IT/FCR3	D517 – L1024	58.4	-	nt
var2CSA	DBL2	IT/FCR3	G550 – L1024	54.6	-	nt
var2CSA	DBL2	IT/FCR3	D545 – L1024	55.2	-	nt
var2CSA	DBL2-ID2	IT/FCR3	D517 – G1229	81.6	-	nt
var2CSA	ID1-DBL2-ID2	IT/FCR3	V490 – G1229	84.4	-	nt
var2CSA	ID2	IT/FCR3	V878 – K1201	38.4	-	nt
var2CSA	ID2	IT/FCR3	S843 – G1229	45.2	-	nt
var2CSA	ID2	IT/FCR3	L1024 – G 1229	24.9	-	nt
var2CSA	ID2-DBL3	IT/FCR3	N 859 – C 1576	83.0	-	nt
var2CSA	DBL3	IT/FCR3 ^3^	L1221 – E1541	37.3	negligible	yes
var2CSA	DBL3	IT/FCR3	C1202 – C1576	43.8	+	yes
var2CSA	DBL3	3D7	C1224 – Y1510	33.2	-	nt
var2CSA	DBL3	Dd2	C1219 – Y1529	44.2	-	nt
var2CSA	DBL3	Pc49	C1227 – Y1540	43.8	-	nt
var2CSA	DBL3	7G8	C1199 – E1587	45.0	++	nt
var2CSA	DBL4	IT/FCR3 ^3^	S1594 – V1888	34.7	++	no^4^
var2CSA	DBL4-DBL5	IT/FCR3	G1588 – E2288	85.4	-	nt
var2CSA	DBL4-DBL5	IT/FCR3	S1594 – E2288	82.5	-	nt
var2CSA	DBL4-ID4-DBL5	IT/FCR3	S1594 – R2271	66.8	-	nt
var2CSA	DBL4-DBL5	3D7	S1576 – T2291	85.6	-	nt
var2CSA	DBL4-DBL5	3D7	G1550 – T2291	88.3	-	nt
var2CSA	DBL4-DBL5	7G8	S1604 – T2318	89.2	-	nt
var2CSA	DBL4-DBL5	7G8	G1578 – T2318	88.3	+	nt
var2CSA	DBL5	IT/FCR3 ^3^	L2003 – L2270	31.7	-	nt
var2CSA	DBL5	IT/FCR3	N1893 – N2290	46.6	-	nt
var2CSA	DBL5	IT/FCR3	K1984 – E2288	36.6	+	yes
var2CSA	DBL5	IT/FCR3	E1971 – N2290	37.8	-	nt
var2CSA	DBL5	7G8	Q2000 – T2318	38.8	++	yes
var2CSA	DBL5	3D7	L1997 – A2269	32.2	-	nt
var2CSA	DBL5	3D7	K1978 – T2291	37.9	-	nt
var2CSA	DBL5	3D7	L1997 – T2291	35.8	-	nt
var2CSA	DBL5	3D7	E1964 – T2291	39.6	-	nt
var2CSA	DBL5	3D7	K1923 – T2291	44.6	-	nt
var2CSA	DBL5	3D7	N1888 – T2291	48.3	+	yes
var2CSA	DBL6	IT/FCR3 ^3^	E2322 – E2590	32.7	++	yes

*P. pastoris *clones were grown overnight at 0.9 L to build biomass and protein induced at 0.3 L scale. Yields following a single nickel chromatography step; – (not detected), + (< 1 mg/L), ++ (1 mg/L). nt, not tested.

^1 ^Molecular weight predicted by Vector NTI software.

^2 ^Rabbit antibodies were screened against CSA-binding infected erythrocytes and negative control A4ultra-infected erythrocytes expressing a non-VAR2CSA protein

^3 ^Data previously published by [18].

^4 ^Antibody reactivity could be revealed by pre-treating IEs with chymotrypsin or trypsin

Secretion of IT4-DBL2 could also be improved by extending construct boundaries by 2 cysteine residues (D517–D833) (Figure 3), but these two additional cysteines were not part of a CX_1–2_C motif (Figure 1) and longer constructs including the predicted CX_1–2_C motif (D517-L1024 and G550-L1024) were not secreted. Because the interdomain region following DBL2 has a number of cysteine residues and it is not proven where the CIDR-like fold begins, we investigated several different construct boundaries to improve DBL2 production. Altogether, seven different DBL2 constructs were tested, but only two were weakly secreted (Figures 3, Table 1). Four different versions of the ID2 region were also examined but none of these were secreted (Figure 3, Table 1). Therefore, two additional cysteine residues at the C-terminus improved IT4-DBL2 secretion, but constructs containing the entire predicted S3 subdomain were not secreted.

For DBL5, the original IT4-DBL5 construct L2003–L2270 was not secreted [18], but a longer K1984-E2288 construct that included predicted C11 and C12 cysteines was secreted (Figure 3). In contrast to IT-DBL5, a shorter IT4-DBL4 recombinant protein lacking the predicted full-length domain was highly secreted [18]. To investigate whether a highly-expressed domain could 'rescue' expression of a poorly secreted domain, several different IT4 DBL4-DBL5 tandem domain construct boundaries were tested, but none were secreted even though they included the longer C-terminus that improved DBL5 secretion (Figure 3). In contrast, one of two different DBL4-DBL5 tandems from the 7G8 VAR2CSA allele was weakly secreted, but neither 3D7 DBL4-DBL5 tandem was secreted. The 7G8 DBL4-DBL5 protein was partially trunctated (Figure 4).

As longer construct boundaries allowed IT-DBL5 to be secreted, the equivalent boundaries were investigated in two other VAR2CSA alleles. Similar to IT4-DBL5, the predicted full-length 7G8-DBL5 recombinant protein was highly secreted (1.3 mg/L) (Table 1). However, the same C-terminal boundaries did not work for 3D7-DBL5 unless the N-terminal boundary was also extended (Figure 3, Table 1). A total of five different N-terminal boundaries were tested for 3D7-DBL5, but only N1888-T2291 was secreted (Figure 3). Interestingly, the secreted 3D7-DBL5 was smaller than expected (Figure 4). Edman degradation analysis indicated that the majority of 3D7-DBL5 recombinant protein was cleaved at position 95 (D1983), accounting for the decrease in molecular weight. After cleavage, the truncated 3D7-DBL5 protein had very similar boundaries to IT4-DBL5 and 7G8-DBL5 and was only slightly larger than one of the 3D7-DBL5 constructs that was not secreted. These findings suggest that longer construct boundaries could improve IT4-DBL5 production, but that *P. pastoris *secretion was sensitive to N-terminal domain boundaries and partially sequence context dependent.

Since longer C-terminal domain boundaries improved the yield of three poorly or non-secreted VAR2CSA domains, longer construct boundaries were also examined for the DBL1 domain, which was highly secreted even though it lacked the predicted C11 and C12 cysteine residues [18]. Predicted full-length versions of VAR2CSA DBL1 domain from the 3D7, 7G8, and Dd2 parasite strains were highly secreted by *P. pastoris *with yields exceeding 1 mg/L (Table 1). Under non-reducing conditions, the majority of recombinant protein in each case appeared to run as a monomer, although there was some higher, dimeric-sized protein forms that were generally lost after reduction (Figure 4). Taken together, these results indicate that C11 and C12 residues are not essential for recombinant protein secretion in *P. pastoris*, but that extending the N- and C-terminus to include extra cysteines residues believed to be important in domain folding improved production of several poorly or non-secreted VAR2CSA DBL domains and also worked for a highly secreted domain.

### *Pichia pastoris *culture adjustment to improve yield

In addition to modifying domain boundaries, protein yield may also be improved by optimizing culture conditions during shake flask fermentation. Protein yield has been found to be affected by duration of methanol induction (2–5 days) and methanol concentration between 0.5% and 5.0% [32]. Whereas there are trade-offs with longer protein inductions because unstable proteins may be more susceptible to degradation and higher alcohol concentrations can kill *P. pastoris*, optimized DBL construct boundaries may enhance recombinant protein stability and permit longer inductions. For these studies, transgene expression was induced for different lengths of time and methanol concentrations. Under standard conditions, 48 h of induction with 0.5% methanol, 7G8-DBL5 protein was highly expressed (>1 mg/L) with no improvement after longer periods of induction (Table 2). However, for several poorly expressed proteins, IT4-DBL3, 3D7-DBL5, and 7G8 DBL4-DBL5, longer inductions significantly improved protein yield (Table 2). Although longer inductions were not able to rescue secretion of all VAR2CSA constructs, similar improvements were also observed for two DBL domains from non-VAR2CSA proteins (Table 2). Conversely, higher concentrations of methanol (3%) did not improve protein production. Overall, optimal culture condition for VAR2CSA protein secretion seemed to be 120 h at a methanol concentration of 0.5–1%.

**Table 2:** Effect of methanol concentration and induction time on recombinant protein yield

		Methanol concentration and induction time		
		
		0.50%		1%
		
Construct		48 h	120 h	120 h
var2csa 7G8 DBL5	Q2000-T2318	1.2 mg/L	0.9 mg/L	Nt
var2csa IT DBL2-ID2	D517-G1229	0	0	0
var2csa IT DBL3	L1221-E1541	0.1 mg/L	0.375 mg/L	1 mg/L
var2csa 3D7 DBL5	N1888-T2291	0.21 mg/L	0.46 mg/L	Nt
var2csa 7G8 DBL4-DBL5	G1578-2318	0	0.1 mg/L	Nt
var22 IT DBL3	G1180-N1488	0	nt	0.75 mg/L
var18 IT DBL3	N1247-D1545	0	nt	0.125 mg/L

*P. pastoris *clones were grown overnight at 0.9 L to build biomass and protein was induced at 0.3 L scale at different durations and methanol concentrations. nt, not tested.

### *Pichia pastoris *recombinant proteins express native protein epitopes

To test whether secreted proteins express native protein epitopes, rabbits were immunized with eleven of the fifteen purified VAR2CSA recombinant proteins (Figure 4). For every protein except IT4-DBL4, immune sera reacted with the surface of CSA-binding infected erythrocytes by flow cytometry, but not with a negative control non-CSA-binding infected erythrocyte (Table 1), indicating that *P. pastoris *expressed VAR2CSA recombinant proteins possessed native epitopes. In addition, four of these recombinant proteins were previously shown to react in a gender-specific manner with malaria endemic sera [18]. These findings suggest that recombinant DBL domains secreted from *P. pastoris *can be used for studying immune acquisition in pregnant women and vaccine development.

## Conclusion

VAR2CSA is the primary candidate for a pregnancy malaria vaccine, but a fundamental challenge for vaccine development is identifying parts of this large protein that are targets of a protective immune response and that are amenable to heterologous production of the disulfide-rich DBL domains. Previous genome-scale attempts to express recombinant *Plasmodium *proteins in *E. coli *for X-ray crystallography have primarily focused on non-membrane proteins that lacked disulfide bonds and the success rates for soluble proteins were relatively low [33,34]. While significant progress has been made to refold DBL recombinant proteins from *E. coli *or to secrete VAR2CSA recombinant proteins from eukaryotic expression systems [18-24], some VAR2CSA domains have proven more challenging to produce than others and only a subset of domains have been expressed in *P. pastoris *[18] or as refolded proteins from *E. coli *[21]. Furthermore, new structural information on the disulfide bonds in DBL domains suggests that PfEMP1-type DBL domains may be larger than previously predicted by sequence homology, but there have been no systematic attempts to optimize construct boundaries for protein secretion. The inability to produce large amounts of all VAR2CSA domains has hindered investigation into PAM immunity and vaccine development.

In this study, new structural information on the disulfide bonds in DBL domains was used to revise domain boundaries in VAR2CSA and the new boundaries were experimentally investigated for recombinant protein expression in *P. pastoris*. Based on sequence comparisons, VAR2CSA DBL domains contain many of the conserved cysteine residues present in solved DBL domains and would be expected to make many of the same disulfide bonds. This prediction is also supported by recent homology models of the individual VAR2CSA DBL domains that came to similar conclusions about domain boundaries and disulfide bonds [35]. Although there is strong support from sequence comparisons and homology modeling [35] to modify the domain boundaries in VAR2CSA, longer construct boundaries including these amino acids were not always secreted from *P. pastoris*. Overall, extending construct boundaries to include the predicted C11 and C12 cysteines generally improved protein secretion and allowed production of previously poorly or non-secreted DBL domains. However, protein secretion was variable, in that slight difference in domain boundaries affected protein production and construct boundaries that worked well for one VAR2CSA allele did not necessarily apply to other alleles. Furthermore, some VAR2CSA regions, such as DBL2 or ID2 were more difficult to clone into bacteria or express from *P. pastoris*. Altogether 15 of 42 (36%) VAR2CSA constructs were secreted from *P. pastoris *and the new construct boundaries allowed production of all six VAR2CSA DBL domains. These will provide new tools to investigate PAM immunity. Although larger proteins, including the tandem DBL domains from EBA175 have been produced in *P. pastoris *[17], size appeared to influence protein secretion. 14 of the 15 secreted proteins were single domains between 30–40 kDa, but only one of nine constructs greater than 70 kDa was secreted. In conclusion, these findings are consistent with recent structural and functional studies supporting a revision of DBL boundaries in PfEMP1 proteins and have application for placental malaria vaccine development.

## Competing interests

The authors declare that they have no competing interests.

## Authors' contributions

MA designed the study and carried out parasite work, protein expression in *P. pastoris*, and flow cytometry. MH, MC, and SG performed cloning and expression of proteins in *P. pastoris*. DLN performed recombinant protein characterization. JDS designed the study, analysed data, and wrote the manuscript with MA. All authors read and approved the final manuscript.
